# Uptake of, barriers and enablers to the utilization of postnatal care services in Thyolo, Malawi

**DOI:** 10.1186/s12884-023-05587-5

**Published:** 2023-04-19

**Authors:** Alinane Linda Nyondo-Mipando, Marumbo Chirwa, Andrew Kumitawa, Sangwani Salimu, Jacqueline Chinkonde, Tiyese Jean Chimuna, Martin Dohlsten, Bongani Chikwapulo, Mesfin Senbete, Fatima Gohar, Tedbabe D. Hailegebriel, Debra Jackson

**Affiliations:** 1 Department of Health Systems and Policy, School of Global and Public Health, Kamuzu University of Health Sciences, Blantyre, Malawi; 2 Maternal and Fetal Health Group, Malawi Liverpool Wellcome Programme, Blantyre, Malawi; 3 Department of Epidemiology and Statistics, School of Global and Public Health, Kamuzu University of Health Sciences, Blantyre, Malawi; 4 UNICEF Malawi Country Office, Lilongwe, Malawi; 5 Department of Maternal, Newborn, Child and Adolescent Health and Ageing, World Health Organization, Geneva, Switzerland; 6 Quality Management Directorate, Ministry of Health, Lilongwe, Malawi; 7 Health Section, Programme Division, UNICEF Headquarters, New York, NY USA; 8 London School of Hygiene and Tropical Medicine, London, UK; 9 University of the Western Cape, Cape Town, South Africa

**Keywords:** Postnatal care, Neonates, Male involvement, Community support, Mothers

## Abstract

**Background:**

Postnatal care (PNC) ensures early assessments for danger signs during the postpartum period and is to be provided within 24 h of birth, 48–72 h, 7–14 days, and six weeks after birth. This study assessed the uptake of and the barriers and facilitators to receiving PNC care among mothers and babies.

**Methods:**

A concurrent mixed-method study employing a retrospective register review and a qualitative descriptive study was conducted in Thyolo from July to December 2020. Postnatal registers of 2019 were reviewed to estimate the proportion of mothers and newborns that received PNC respectively. Focus Group Discussions (FGDs) among postnatal mothers, men, health care workers, and elderly women and in-depth interviews with midwives, and key health care workers were conducted to explore the barriers and enablers to PNC. Observations of the services that mothers and babies received within 24 h of birth, at 48–72 h, 7–14 days, and six weeks after birth were conducted. Descriptive statistics were tabulated for the quantitative data using Stata while the qualitative data were managed using NVivo and analysed following a thematic approach.

**Results:**

The uptake of PNC services was at 90.5%, 30.2%, and 6.1% among women and 96.5%, 78.8%, and 13.7% among babies within 48 h of birth, 3 to 7 and 8 to 42 days respectively. The barriers to PNC services included the absence of a baby or mother, limited understanding of PNC services, lack of male involvement, and economic challenges. Cultural and religious beliefs, advice from community members, community activities, distance, lack of resources, and poor attitude of health care workers also impeded the utilisation of PNC services. The enablers included the mother’s level of education, awareness of the services, economic resources, community-based health support, adequacy and attitude of health workers, seeking treatment for other conditions, and other clinic activities.

**Conclusion:**

Optimisation of uptake and utilization of PNC services for mothers and neonates will require the involvement of all stakeholders. The success of PNC services lies in the communities, health services, and mothers understanding the relevance, time points, and services that need to be delivered to create demand for the services. There is a need to assess the contextual factors for a better response in improving the uptake of PNC services and in turn inform the development of strategies for optimizing the uptake of PNC services.

**Supplementary Information:**

The online version contains supplementary material available at 10.1186/s12884-023-05587-5.

## Background

The World Health Organisation (WHO) recommends the provision of Postnatal Care (PNC) to the mother and neonate within 24 h of birth irrespective of where the birth occurred and also at 48–72 h, 7–14 days, and six weeks after birth [1]. The WHO asserts that postnatal care is a critical component of care among mothers and their babies because it is the bedrock for the provision of the cascade of care which encompasses the provision of promotive and preventive care, detection of early problems, and curative services to the pair within the first 6 weeks of delivery [1]. Early postnatal care ensures initial assessments for danger signs during the postpartum period such as excessive bleeding in the mother and other neonatal problems. Improving the quality of care provided during the postpartum period increases the utilization of the services [2].

Despite recognizing the relevance of PNC care, its coverage remains low worldwide along the continuum of care with 71% and 64% of women and newborns accessing the services as of 2020 [3]. A Sub-Saharan systematic review reported a pooled utilization of PNC rate of 52.48% with disparities between those that dwelled in rural areas at 3.38 and their urban counterparts at 36.66 [4]. The same low trends are prevalent in Malawi where despite the steady progress in achieving higher rates of women accessing antenatal care (ANC) (97%) and delivery care (97%), the coverage of PNC care for mothers and newborns within the first 48 h of delivery is at 84% and 88% respectively [5]**.**

Postnatal care in Malawi is offered within primary and secondary care facilities with the tertiary facilities offering mostly in-hospital services. Malawi’s guidelines recommend the provision of PNC at 3 time points; first, within 2 days in a health facility following delivery, second visit within one week after delivery, and the third visit at 6 weeks which is also the end of puerperium and [5]. The guidelines recommend that Health Surveillance Assistants (HSAs) provide postnatal care services at home-follow ups in their respective communities [5]. Malawi adopted the WHO guidelines on the services and assessments that women and their newborns should receive during postnatal care visits [6]. Assessment for the mothers includes amount and flow of vaginal bleeding, uterine contraction, fundal height, temperature, and pulse within 24 h after birth [1]. In the subsequent PNC assessments, mothers are asked about micturition, urinary incontinence, bowel function, healing of a perineal tear, headache, fatigue, backache, perineal pain and hygiene, breast and uterine tenderness, and lochia. On each visit breastfeeding process, emotional well-being and support women receive postnatally are assessed and women and family members are encouraged to report any changes in mood, emotional well-being, and behaviour that is inconsistent with the woman [1]. The information shared with a woman is on family planning methods and signs of postpartum heamorrage [1]. At each postnatal visit, a newborn is assessed if: stopped feeding well, if any reported history of convulsions, breathing rate of more than 60 breaths/minute, presence of severe chest indrawing, absence of spontaneous movements, the temperature of more than 37.5 degrees Celsius, a body temperature of less than 35.5 degrees Celsius, any signs of jaundice in the first 24 h, yellow palms and soles at any age [1].

There is limited research in Malawi on PNC services with more maternity studies focusing on antepartum and intrapartum services. Evidence from earlier studies shows that midwives rarely conduct postnatal checks on mothers without known complications unlike those that presented with a risk factor or complaint due to the pressure of work [7] and were regarded as substandard with gaps in monitoring [7, 8]. A Sub-Saharan systematic review showed that there were disparities in PNC utilization and argued for improvements in the quality of PNC services provided in the region [4]. The evident disparities in utilization call for an understanding of barriers and enablers to PNC utilization in a Malawian setting. Given the paucity of data on postnatal care in Malawi, this study assessed the uptake of postnatal care among mothers and babies and the barriers and facilitators to receiving that care. This information will be relevant for use by policymakers both locally and beyond in strengthening the delivery and uptake of PNC. This information will also respond to the call to strengthen the delivery of postnatal care as outlined in the 2023–2030 Health Sector Strategic Plan III for Malawi [9]. Postnatal care was also an emphasis in the sexual and reproductive health and rights policy for Malawi [6].

### Study design

This was a mixed-method study using a concurrent triangulation design [10, 11] that was conducted from July to December 2020 in Thyolo District, Malawi. A concurrent triangulation design aims at corroborating and cross-validating findings by using both qualitative and quantitative studies [10]. The data were merged at the point of interpretation to highlight areas of convergence and divergence [10]. The quantitative data was collected from a review of retrospective PNC and Neonatal registers whereas the qualitative data were collected using a descriptive study and non-participant observations as an ethnographic approach.

### Study setting

The study was conducted in three facilities in Thyolo district. Thyolo is in the Southern part of Malawi and has a population of 458,976, and of these 177, 298 are women of childbearing age [12]. The facilities included a health centre located at a trading centre with vibrant economic activities 20kms from the district hospital (facility 1), a rural-based hospital located 53kms from the District Hospital (facility 2), and the district hospital (facility 3) which is also the referral hospital for all health centres and hospitals in the district. The selection of sites was purposive to maximize the breadth of responses from the qualitative component and to achieve representation of the district from the retrospective review of registers. The study was conducted in Thyolo because it is one of the districts with poor maternal and newborn institutional indicators with the maternal mortality ratio and neonatal mortality rate in 2017/2018 at 136/100,000 live births and 27/1000 live births respectively [13]. All facilities provide maternity services with only the district hospital conducting caesarian sections.

### Sampling and sample size

#### Quantitative retrospective audit sampling and sample size

The sample size was calculated using Cochran’s sample size calculation formula [14]. In 2019, the number of postnatal women registered in Thyolo was 4328, 664 were from facility 1, 810 were from facility 2, and 2854 were from facility 3 [13]. In the same year, there were 4382 neonates in the register with 669 from facility 1, 821 from facility 2, and 2892 from facility 3 [14]. We employed a proportionate sampling technique and aimed at 95% CI, a margin error of 5%, and a population proportion of 50%. Using the above estimates the sample size was 353 women and 354 neonates. The study followed a systematic random sampling [15] and used PNC registers for 2019 in the three facilities because they were the most recent ones at the time of the study. A random start point was selected from a box of random numbers, and it was between serial numbers 1 and 10.

#### Qualitative sampling and sample size

We used a purposive approach with maximum variation [16] and included participants of varying characteristics to broaden the scope of our responses. The sampling frame and sample size are illustrated in Table 1.

**Table 1:** Sample size and sampling technique for qualitative component

**Participant type**	**Method of data collection**	**Number**	**Sampling Technique**	**Rationale**
Maternity Staff	In-depth Interview	7	Purposive	These were key to the provision of PNC services at various levels
Elderly women and Grandmothers	Focus Group Discussions	24	Purposive	They advise women on maternity aspects
Men	Focus Group Discussions	24	Purposive	They are decision-makers in families and provide for resources to access care
Health Surveillance Assistants	Focus Group Discussions	24	Purposive	They provide Postnatal Care services in static and outreach centres. They are liaison points with the community
Postnatal Mothers	Focus Group Discussions	24 (Varied with age)	Purposive	These are the beneficiaries of the services

### Data collection

#### Retrospective register review

A data extraction form that mirrored components of the registers was used to extract data from the PNC registers (Additional Files 1 and 2). The tool was assessed for face validity by a group of experts in maternal and neonatal care and checked against the register. Data were extracted by three trained Research Assistants. Postnatal registers from January to December 2019 were reviewed to estimate the proportion of mothers and newborns that received any PNC and the components of the services they received.

#### Focus group discussions and in-depth interviews

Qualitative data were collected using Focus Group Discussions (FGDs) among postnatal mothers, men whose partners were postnatal mothers, health care workers, and elderly women and grandmothers. We conducted in-depth interviews with midwives and key healthcare workers. Focus Group Discussions were deliberated in Chichewa (local language) using discussion guides (Additional Files 3 and 4) that were pretested before data collection. The results of these pilot interviews were not incorporated in the findings however they assisted in revising the questions for clarity and incorporation of additional probes. Data were collected by four trained research assistants that are well-versed in qualitative research. All participants were identified with pseudo names during the FGDs and each one was asked to identify themselves with the pseudo name before contributing during the FGDs. FGDs were conducted in a private setting as selected by the participants. Interviews among healthcare workers were conducted in Chichewa following an interview guide (Additional File 5). After each interview and discussion, research assistants summarized key findings and shared them with the participants as a form of member checking [17].

##### Quality of qualitative data

Our results include the quotes of the participants to increase the dependability of our findings that they are founded in what the participants stated [17]. We achieved credibility of our findings by collecting data from multiple sources such as healthcare workers, postnatal women, elderly women, and male partners and using multiple methods of data collection such as FGDs, observations, and in-depth interviews [17]. To adequately address reflexivity, the research team reflected on their experiences and knowledge of postnatal care services to avoid their experiences from guiding the data collection and influencing the analysis of the data [18]. This was achieved through team meetings during the period of data collection which was also supported by each researcher writing down their experiences, assumptions, and knowledge regarding postnatal care [18]. Team meetings were an iterative process during the data collection and analysis phase [18].

#### Non-participant observations methods

We observed the PNC services that mothers and babies received within 24 h of birth, at 48–72 h, 7–14 days, and six weeks after birth. We conducted observations for a total of 36 h in the three facilities during the day, night, and early morning shifts dependent on the specific time point and availability of a participant. We observed a postnatal woman with or without a living baby, varied ages of babies including 24 h, 48 h, 7 days, and 42 days after birth, and had to be 16 years of age and above. We focused on interactions between postnatal women, and their babies with health care workers and paid attention to the health services they received and anything else of note to the experience of PNC (Additional File 6 Observation Checklist).

#### Data management and analysis

All data were kept in locked cabinets at Kamuzu University of Health Sciences (KUHeS) and in password-protected computers with access limited to lead researchers. Quantitative data from the registers were entered into an excel database that was later exported to Stata for descriptive analysis where frequencies were tabulated. We calculated the frequencies of postnatal care services such as Family planning, HIV services, Receipt of Vitamin A, K, and tetracycline eye ointment, low birth weight, and cord care. We could not determine associations because the registers do not capture sociodemographic details that are a prerequisite for such analysis.

Audio recordings were transcribed verbatim, translated into English as applicable, and managed using NVivo 12 software (QSR International, Melbourne, Australia). Data were analysed using a thematic approach following a coding framework that was developed from a review of the initial transcripts [19]. The team iteratively discussed the findings as the data collection and analysis progressed. All similar codes were grouped under overarching themes of either barriers or facilitators. Each overarching theme was later classified to show the factors at different levels of individuals, health systems, and communities. All tenets under themes were verified against the audio to ensure that they were representative of the data.

## Results

### Quantitative results

We reviewed entries of 358 mothers and 358 entries of newborns. Among these women, 65.1% were from facility 3, 17.9% from facility 2, and 17% from facility 1. Of these women, 5.1% were HIV positive, and one woman died (Table 2). Access to PNC by mothers was 90.5% within 48 h, 30.2% from 3 to 7 days period, and 6.1% from 8 to 42 days period. The postnatal care services that were provided included uterine assessments, assessments for complications, and the provision of family planning methods. The majority of the women (99.2%) had an involuted uterus, none of the women had postnatal complications, 77.2% of the women were advised about family planning and all the women breastfed their babies. Vitamin A was administered in 20.1% of the cases and 12.1% were on family planning methods postnatally (Table 2). All women had normal lochia and intact episiotomy.

**Table 2:** Summary of maternal post natal care register *N* = 358

**Hospital**	**Participants**	**Percent**
Mothers’ status		
Alive	357	99.7
HIV test		
Positive	17	5.1
Post Natal Care		
Family Planning Care		
No	80	22.3
Post Natal HIV test		
Negative	72	98.6
Vitamin A		
Yes	58	20.1
No	230	79.9
Post-Natal Family Planning		
Yes	16	12.8
No	109	87.2

### Summary of findings from neonatal registers

A total of 358 entries of babies were reviewed. Among these babies, 65.1% were from facility 3, 17.9% from facility 2, and 17% from facility 1. The average birth weight for babies was 3.0 kg with a standard deviation of 0.4 kg. The majority of babies were alive (98.2%) and some of the neonates developed complications at birth with 14.8% low birth weight, 2.2% developed asphyxia and 3.7% were resuscitated (Table 3). 96.5% of the babies accessed PNC within 48 h, 78.8% of the babies accessed PNC between 3 to 7 days and 13.7% of the babies accessed PNC from 8 to 42 days. Of the post-natal care received, 93.5% received tetracycline eye ointment, 4.8% were HIV exposed and received Nevirapine and 9.4% received cord care (Table 3).

**Table 3:** Summary of findings from the neonatal registers

Hospital	Participants	Percent
Asphyxiated- Resuscitation		
No	289	96.3
Baby status		
Died	3	0.8
Post Natal care		
Low birth weight		
No	308	86.5
Immunization		
BCG & POLIO	332	95.4
No	16	4.6
Tetracycline Eye Ointment		
Yes	319	93.5
No	22	6.5
Cord care		
No	308	90.6
Vitamin K		
Yes	24	7.1
No	312	92.9
HIV exposed Infant nevirapine		
No	280	95.2

### Characteristics of qualitative participants

We had 3 FGDs with 24 Health Surveillance Assistants (HSAs) and of these 12 were females. All of them had attained a secondary school education with 16 having attained Malawi School Certificate of Education (MSCE) and the others having a Junior Certificate of Education (JCE). Their years of service ranged from 6 to 27 years and their age ranged from 23 to 49 years old. Each FGD comprised 8 HSAs.

We interviewed 7 Nurses/Midwives and of these 4 were Registered Nurses Midwives. Five of these were either facility or Departmental In-charges. The range of service was from 6 months to 9 years. Six were female and the age ranged from 24 to 36 years.

The demographic characteristics of postnatal women, men, and elderly women are presented in Table 4.

**Table 4:** Demographic characteristics of postnatal women, men, and elderly women *N* = 72

**Variable**	**PNC women ** ***n*** ** = 24**	**Men ** ***n*** ** = 24**	**Elderly Women ** ***n*** ** = 24**
Age range	18–36	28–72	42–73
Employment			
Housewives	9	0	0
Businesses	7	14	6
Farmers	5	7	18
Formerly employed	2	3	0
Health Volunteer	1	0	0
Education			
None	0	0	3
Primary	9	16	21
Secondary	13	8	0
College	2	0	0

### Barriers and facilitators to postnatal care services attendance

The barriers and facilitators that influence attendance to PNC services in Thyolo have been categorized under three levels, individual, community, and health systems. This classification is not rigid as the factors are interrelated but have been selected to promote ease of understanding (Table 5).

**Table 5:** Barriers and enablers to attendance to postnatal care services in thyolo

**Factors that Influence attendance to Postnatal Care Services in Thyolo**		
**Theme**	**Barriers**	**Facilitators**
**Individual Level**	Absence of a baby or mother: Relocation or deaths	Level of Education
	Inadequate Understanding of PNC services	Recognition That PNC is a mother’s responsibility
	Lack of Male Involvement	Male Involvement
	Economic Activities and Factors	Economic Resources
**Community Level Barriers**	Cultural and Religious Beliefs	Community-based health support
	Advice from Community Members	Community Awareness
	Community Activities	Advice from Elderly women and Peers
	Distance	Adequacy and attitude of Health workers
**Health System Barriers**	Resources	Seeking treatment for other conditions
	The attitude of healthcare workers	Clinic activities and operations

### Individual level barriers

The individual-level barriers included the absence of a baby or mother secondary to the relocation of the mother-infant pair or death, inadequate understanding of PNC services, lack of male involvement, and economic factors.

#### Absence of a mother-infant pair: relocation of mother-infant pairs or death of either mother or infant

Participants that relocate from one area to another find it hard to initiate or continue with PNC services, especially in the absence of a proper transfer. Participants stated that this was common with women and babies that come from bordering countries like Mozambique secondary to porous borders with Malawi. Once they deliver within facilities in Thyolo, they no longer report for postnatal services.

*“Others happen to come here to give birth, but they live in Mozambique [country] so they do not have time to come back here to the hospital for post-natal”* HSA’s FGD Facility 2

It was noted that the babies whose mothers died or those whose mothers had suffered a mental illness were less likely to continue with PNC. If they continue, then it is sporadic because in most cases they are cared for by the grandmothers who are usually old and cannot manage to keep up with postnatal visits.

*“I have seen several women who are mentally ill. These women and their children do not come for post-natal following the proper procedure. The orphans also do not come procedurally because the people who look after them, are reluctant.”* Health Worker, in-depth interview, Facility 1

Mothers that have lost their babies rarely attend PNC services as they are either self-discriminating or at times find that the services have no targeted agenda for them. Again, in the communities, they are discouraged to attend the services as there is a prevailing belief that the services are for those with babies.

*“… They [Mothers without babies] feel like coming here for post-natal at seven days and after six weeks, is good when the baby is there when the rule is not like that. Whether they feel pity that they will be seeing their friends have babies in their arms when they do not have babies. So, they just decide to stop that they should not come.”* Health Worker, in-depth interview, Facility 1

### Inadequate understanding of PNC services

The lack or limited understanding of the relevance of PNC bars the women from attending the services. Healthcare workers attributed limited understanding to the low levels of literacy among most women that attend PNC. They argued that if the literacy levels were high most women would appreciate the relevance of PNC and that low literacy levels influence their beliefs and use of traditional medicines. Health workers stated that when advised on PNC some women openly state that they will use traditional approaches.

*“Lack of knowledge, like in the case that the person was pregnant and has delivered but they lack knowledge that they are supposed to come with the baby for postnatal despite that they have delivered, it might be that this kind of knowledge has not yet reached this mother…Lack of civic education, maybe the mother was not taught which can make her fail to come.”* HSA’s FGD, Facility 2

### Lack of male involvement

The lack of male involvement in aspects of maternity services including postnatal care limits a woman and her baby’s attendance at PNC. Postnatal women stated that in some instances some husbands bar their women from leaving their homes and would rather have them at home all the time. The belief that the decision-maker in a home is a man influences attendance to PNC by the mother and the baby. In instances when the man is not home then a woman may fail or delay initiating health-seeking behaviour until her partner has consented to that.

*“Sometimes as you know women follow what husbands dictate, when he says the baby must not be helped, as a breadwinner and a decision-maker everyone follows that. As a result, the baby is left in the hands of the grandmother who is not capable of looking after the baby.”* Elderly Women’s FGD, Facility 2

Men corroborated the findings and stated that in some instances men fail to support their partners including a lack of interest in the services that a mother and baby are accessing.

*“Sometimes it is us, men when we choose not to take part in postnatal care. As he has already said, sometimes we fail to provide soap to the mother which makes the woman fail to patronize postnatal care. Sometimes when she goes for postnatal care, we don’t ask her how much the child weighed, and the next date of appointment, as such, she takes postnatal care as if it’s just for her and she has the freedom to go or not.”* Men’s FGD, Facility 2

### Economic activities and factors

The pursuit of activities for economic benefits prevents women and babies from attending PNC. Participants stated that some women would rather attend to their businesses, farming, or other economically rewarding activities than attend to PNC.

*“Social-economic status affects post-natal checkups so much here because other people go for piece work to get money and sustain themselves and miss post-natal. I also mentioned rain, even this morning you cannot see lots of people here because they have gone to their fields since it is growing season.”* Health Worker, in-depth Interview, Facility 2

The lack of finances to support transportation costs also leads to the non-attendance of PNC. Furthermore, the inability to provide supplementary feeds to the baby while at the clinic persuade women to miss PNC appointments. At times women prioritize providing a meal for their family overusing the money to meet transportation costs. They weigh whether to use the available money on transport to the health facility or on food to feed their families.

*“Sometimes transport becomes a challenge. Some of us have to travel by public transport and when it happens that on the specified date we were given, we have no money, we will not go and we start searching for transport… it might be due to lack of money by the guardian.”* PNC Mothers’ FGD, Facility 1

### Community level barriers

The community-level barriers included cultural and religious beliefs, advice from community members, and the various community factors prevalent in a setting.

### Cultural and religious beliefs

Some religious beliefs bar their members from accessing health services from a facility because that would be a sign of disbelief in the religion and the healing powers therein. Women further expressed that even after being visited by HSAs, the women whose religion prohibits them from accessing health services never show up for PNC. Traditional leaders have tried to intervene but have not yielded positive outcomes from some of the religious groups that are against seeking health services and such women also deliver at home.

*“Sometimes it is because of beliefs that some people hold. You see last month I visited some women worshiping with Zion church, I found them pregnant, and I was asking them about antenatal care but only to be told there were praying and when the time comes for delivery, they will do it at home. Such incidents discourage HSAs from going there again and doing the expected work.”* HSA’s FGD, Facility 3

Some cultural beliefs or norms of the management of a baby after delivery prevent mothers from accessing PNC services. Some practices involve elderly women attending to the newborn with herbs that are believed to facilitate the drying of the umbilical cord stump.

*“On the beliefs, usually, when a woman gets discharged after delivery the baby and her mother fall sick when that happens instead of rushing to the hospital, they rush for herbalists that they believe can heal them.”* HSA’s FGD, Facility 3

### Advice from community members

The various pieces of advice that PN women receive from members of their community and the beliefs that are shared across a community and established as norms also contribute to the non-attendance to PNC. Elderly women stated that in some instances older women ill-advised their wards on the importance of PNC, and as such they fail to seek care themselves due to the advice. The advice from the elderly in instances where babies are born at home usually discourages one from attending PNC services which is further compounded by the fear of being penalized for delivering at home.

*“Some women tolerate their children’s laziness and do not take their children to the hospital for postnatal or even when they were pregnant or when they give birth at home. So they are stuck when their babies get sick so they end up going to private hospitals out of fear since they know that at private clinics they will not bother to follow the records because it is a business to them. It is so difficult for the private clinic to diagnose what is wrong with the baby and the baby can die.”* Elderly Women FGD, Facility 1

### Community activities

The activities that a woman engages in within her community may prevent her from attending PNC services. These activities include political rallies, wedding functions, initiation ceremonies, farming, funerals, or any other deemed relevant. In such cases, a woman would opt to attend the events and return to the facility later when she is not occupied.

*“She can tell you that I was busy that is why I failed to come here. Sometimes she will tell you that there was a funeral which I (health worker) feel is part of the negligence because if they can reach a point of telling us that I was busy, ah there was a funeral, had it been that it is not negligence they could have started coming for post-natal for the baby and do the other things later. But it is just negligence.”* Health Worker in-depth Interview, Facility 1

### Health system barriers

The health system barriers included the distance from one’s home to the health facility, inadequacy of resources, and the attitude of health care workers.

### Distance

The distance was of concern among participants from the rural-based health facility and women failed to show up for PNC because they could not walk to the facility which was compounded in cases when they had no finances to cater for their transportation costs.

*“Sometimes some women reside far from the clinic, so it is difficult for them to go every month or every day that was allocated for them to attend antenatal services if the man did not provide transport.”* Men’s FGD Facility 2

### Resources

With the inadequacy of resources in a facility including a shortage of health workers, a facility is unable to reach all those that need to be visited if they do not present themselves at the facility. Women and men also stated that when there is glaring evidence of a shortage of health workers at the various facilities, women would rarely attend postnatal services. The shortage is more apparent in areas where they do not have designated healthcare workers responsible for postnatal services alone like in Health Centres.

*“Sometimes we fail to conduct out-reach clinics because of the lack of transport”* HSA’s FGD Facility 2

*“Sometimes it happens that we are only two midwives, and the labour ward is full rendering us busy, in such cases mothers who are due for discharge complain. Or those who have come for post-natal at seven days and six weeks when they find us busy, they complain.”* Health Worker in-depth Interview, Facility 1

### The attitude of healthcare workers

The attitude of healthcare workers impedes the utilization of postnatal services among women and their babies. Participants stated that the bad attitude includes shouting at the women, manner of reception, conduct while providing care such as time management, and reaction after they show up after missing their scheduled appointment date or if they report during the night. Some of the attitudes that are displayed when a woman is accessing antenatal care services determine if she will use the postnatal services or not. The negative attitude that is displayed by healthcare workers leads participants to conclude that the healthcare workers dislike their work. These sentiments were shared also by healthcare workers.

*“… Sometimes when you go to the hospital after the funeral, some doctors or nurses do not welcome us warmly. With such fear in our minds, we simply skip postnatal care for that month and visit the hospital the following month.”* PNC mothers’ FGD, Facility 1

*“Maybe, the way they speak. Or maybe, unfortunately, let’s say they are late for their visit, and they get shouted at, so some feel discouraged that there they were shouted at leading to embarrassment, especially if in the presence of a group of people. So, some may pull back. But mostly it is about the way they speak.”* Health Worker in-depth Interview, Facility 3.

### Factors that enable utilization of PNC services

The factors were categorized at the individual, community, and health system levels.

### Individual level factors

The individual-level factors encompassed the level of education of a mother, the recognition that PNC is a mother’s responsibility, the availability of economic resources for the money, and the involvement of male partners in PNC.

### Level of education

The level of education facilitates understanding of the relevance of PNC services such that educated women usually attend the services. Participants stated that one’s level of education and understanding facilitates the remembrance of appointment dates for the next visit. In such cases even if a woman has other commitments they can reschedule and attend PNC.

*“Education can help this mother to come for post-natal because that facilitates understanding of issues however when a mother is not educated, it is a challenge for her to understand the relevance and schedule for PNC.”* Health Worker in-depth Interview, Facility 1.

### Recognition that PNC is a mother’s responsibility

Elderly women stated that once women are trained and are aware that it is their responsibility to attend PNC with their babies, they usually abide by such. In agreement, mothers admitted that once are aware of their roles they would prioritize attendance at PNC over other activities.

*“Our responsibility as mothers is to make sure that the child has been given all the vaccines that the doctor prescribed. We should not skip any vaccines because every vaccine that is written in the child's health passport is important.”* PNC Mothers’ FGD, Facility 1

### Economic resources

Availability of finances to support transportation costs or if a family resides close to a health facility, facilitates attendance to PNC. Participants noted that women that have financial capabilities easily attend PNC services. The availability of motorcycle taxis enables women to easily get to a health facility for services.

*“We have motorcycles these days so she can be placed in the middle and us at the back escorting her to the hospital. We will try our best to make it possible for her to go to the hospital. We even use a bicycle to carry her.”* Elderly Women’s FGD, Facility 1

### Male involvement

When men are involved in PNC services and are encouraged to support and accompany their partners and babies for a postnatal check-up, it results in more women and babies attending the services. It was noted that women that attend PNC services with their spouses are prioritized and are attended to first which further motivates women to patronize the services. Men viewed that their involvement lessens transportation challenges that women face which facilitates attendance to PNC services. Men reckoned that their involvement motivates a woman to attend services. Men take note of appointment dates and avail themselves to provide transportation to the facility.

*“What happens is that we carry them on our bicycles from home to the clinic and we get back to business. They cannot walk to the clinic, and if you don’t pick them up, the atmosphere at home will not be good. That encourages the woman because she is assured of the availability of transport. Things are quite easy for those that have a bicycle, but for us who do not have one, we need to work hard to get one so that we should use it to carry them on the day they are supposed to come.”* Men’s FGD, Facility 2

### Community factors

The enabling factors at a community level included: the availability of community-based health support that extends from health surveillance assistants (community health care workers) to the involvement of village health committees reinforcing attendance to PNC services; community awareness platforms on the relevance of PNC; encouragement from the elderly people within a village.

### Community-based health support

The presence of healthcare workers who conduct home visits at the community level facilitates the utilization of PNC services. Their presence in the community lessens the burden that comes with travel for one to get to a health facility. An HSA narrated his plan of work in this manner:

*“Firstly, we register every pregnant mother in the village, once she gets pregnant the HSA must know and have a list of pregnant women, she is visited at least it should not exceed three visits. Once she delivers, she is supposed to notify the HSA that she has delivered so that the baby can also be visited.”* HSA’s FGD, Facility 1

*“We also have some health assistants who stay close to our homes, we can approach them and see how best they can help us.”* PNC Mothers’ FGD, Facility 1

Village health committees assist in encouraging women to attend PNC services. These are established platforms in the community that acts as liaison points between health facilities and the community on health aspects. They notify the health workers should they sense that there is a problem with a particular family that requires health services.

*“Sometimes it is because of the health mentors or assistants who come to our communities to encourage us about going for postnatal, they can even tell us the actual dates when we can go. These mentors are there in almost every village and they advise us on issues to do with safe motherhood.”* Elderly women’s FGD, Facility 1

### Community awareness

Participants stated that there is more awareness now regarding PNC services which encourages utilization. At the community level, the women that fail to show up are also penalized as per local by-laws. It was noted that HSAs usually give health promotion services that equip the communities with the necessary information on PNC services.

*“When we have meetings here in the village, the headmen teach us the importance of reporting for antenatal and postnatal services by both the mother and baby. So they encourage us to report for postnatal care so that all members of our households are healthy too.”* PNC Mother’s FGD, Facility 2

### Advice from elderly women and peers

The pieces of advice that elderly women render to mothers facilitate attendance to PNC services. Elderly women are custodians of tradition and norms related to pregnancy, childbirth, and postnatal care thereby having a greater influence on what a mother does during those times.

*“Sometimes it happens that a mother delivered the baby without any complications and when she goes home, she is faced with another disease, maybe her fellow women will advise her to report back to the hospital for her to be checked.”* Elderly women’s FGD, Facility 2

### Health system factors

The health system factors that enabled the use of PNC services included the human resource factors that encompassed adequacy and attitude; the tendency to seek treatment for other illnesses which enabled them to be at a facility to access PNC services and the activities that a clinic runs that would attract the mother-infant pair as well as the manner of operation at the facility.

### Adequacy and attitude of health workers

When a health facility has adequate staffing levels, it lessens the workload for the other members of staff and promotes the delivery of PNC services which in turn motivates women to attend the services. The availability of community-based health workers eases the burden of long distances to other static facilities because women make use of the available platforms within a village.

*“Having enough health workers in the hospital so that when they come to the hospital they should not be insulted because the doctors speak rudely because they are tired of work so instead of just knocking off when they see a lot of patients, they start speaking rudely some of which I cannot even explain. So, if the doctors will be enough a lot of people will be helped and people will not feel bad about coming to the hospital.”* PNC Mothers’ FGD, Facility 2

A positive, welcoming, and respectful attitude from health workers encourages the utilization of PNC services by mothers and babies. Participants were more concerned with the welcoming attitude of health workers and stated that if they are respected then most of the women will use the services.

*“There are some health workers here who speak to us warmly when we are talking to them so we come here knowing we will not be disappointed in any way.”* PNC Mothers’ FGD, Facility 1

### Seeking treatment for other conditions

The tendency to seek treatment for a condition at a facility following a child’s illness facilitates attendance at PNC because women know that they will be assisted or referred for proper care.

*“Sometimes a woman may not have a plan or appointment to come to the hospital but the baby might get sick or the mother is not feeling well, so she is forced to visit the doctor so that she can be told what is wrong. So, when she reports at a facility she explains her problem, other women may still be bleeding so they come for help.”* Elderly women’s FGD, Facility 1

### Clinic activities and operations

Women get motivated to attend PNC services because of the health promotion activities that take place while at the facility like education, dancing, and singing. The promotion of PNC during the antenatal period also encourages them to attend the services. Women share amongst themselves these activities and others are motivated to attend PNC services. The administration of nutritional support encourages women to attend PNC services. Women recognize clinic activities as beneficial to their babies as they have better health outcomes.

*“It might also be that she is attracted to the teaching on how she can prevent another stillbirth, sometimes they might discover what went wrong for the stillbirth to happen. So, she is advised on what to do to prevent a stillbirth… When you see the progress in the baby’s weight you feel encouraged to be coming for postnatal care services and when you see that there is no progress you still feel motivated to come so that you can be helped.”* PNC Mothers’ FGD, Facility 2

*“The reason which makes the mother have an interest in coming for post-natal with the baby, firstly, is on the part of the hospital… the women who have come for PNC services at seven days after delivery and six weeks are prioritized and attended to first which encourages them to keep coming here at the hospital for they know that they will not wait for a long time.”* Health Worker In-depth Interview, Facility 1

### Results from observations

A total of 11 observations were conducted and covered 1 h, 24 h, 48 h, one week, 2 weeks, 4 weeks, and 6 weeks post-delivery. In total, we observed interactions between postnatal women, and their babies with healthcare workers and paid attention to the health services they received. We observed for a total of 36 h. In summary, the common services that babies received were vaccination with minimal advice on breastfeeding. The women that were due for TTV, received the vaccine. There were minimal instances where physical examinations were performed on the mother and baby and rarely was there any counseling done including among new and young mothers. There were exceptional cases where student nurses were available where education, demonstration of breastfeeding, and examinations were done. There were cases where women were seen in pain after delivery and there was no indication of receipt of painkillers, let alone examination that is needed especially for those that were observed 24 h post-delivery and were about to be discharged. Women would share advice among themselves, or their guardians would advise them and others in the ward on what to do. Some of the pieces of advice included squatting in the delivery room to facilitate the delivery of the placenta and tying a wrapper around the tummy after delivery which is believed to aid in the management of abdominal pains and cleansing of any products of conceptions (Table 6).

**Table 6:** Summary of observations

**Timepoint post-delivery**	**Centre**	**Number of Children**	**Hours of Observations**	**Services received**
1 h	Facility 1	1	5	• Instructed to clean up and bathe • No other services during the 5 h of observation • The nurse would enter the PNC ward and would only attend to those being discharged
24 h	Facility 1	3	4	• HSA administers vaccination • A message inquiring about breastfeeding is broadcasted
24 h	Facility 2	1	3	• No interaction with a health care worker
24 h	Facility 2	2	4	• Seemingly in pain at the time of observations • No Nursing activities were observed • HSA announced of need for Vaccines
48 h	Facility 2	2	3	• HSA announced of need for Vaccines and educates the women • A nurse calls the mother under observation to her office for discharge procedures
1-week PNC check-up	Facility 3	1	4	• Health education by a student nurse on danger signs that the baby and mother may experience during the post-natal period, breastfeeding, and attachment including a demonstration, time points for post-natal check-ups, mentioned one week and 6 weeks and said that at six weeks they should come to start family planning and under-five clinics for the baby, child care and self-care at home, hygiene, keeping their genital area clean and those who had episiotomy should do sitz baths to help with healing of the wound • The nurse also talked about keeping the baby clean and warm all the time • HSA checks her baby's health passport book to ascertain receipt of Vaccines • Another student nurse was checking the temperatures of both mothers and babies, checked on breastfeeding procedures, and made necessary demonstrations • The baby was weighed, asked if breastfeeding and weight were communicated to the mother • Mother had abdominal palpation, asked about bleeding and if in pain, and all recorded in the health passport book and communicated on the date of the next visit at 6 weeks
4 weeks PNC	Facility 2	2	2	• At the time of discharge, there were no vaccines at the facility, she then got sick and got traditional medicines hence reporting 4 weeks later • Mother kept baby covered but baby looked malnourished, • No documentation about labour and delivery in the health passport book • Breastfeeds the baby for a very short time and quickly covers the baby. Poor positioning while breastfeeding • The baby is registered and gets a vaccine, the mother counseled on the vaccine and is informed of where to access the next vaccines and the date
2 months post-delivery	Facility 3	1	3	• Two months old unvaccinated baby, a student nurse alerts another nurse on the vaccination station that the baby will need all vaccines • HSA gives education on vaccines and their schedule and the under-five clinic and asks women to sing an under-five related song • Babies are weighed by student nurses with the help of HSAs and those eligible for vaccines are channeled appropriately • Student nurses demonstrate how to hold the baby but rarely shared what vaccine the baby is receiving • A cotton swab is given out to mothers without any explanation of its use and mothers learn from each other
6 weeks PNC	Facility 1	2	3	• The baby is weighed by HSA • HSA asks for the baby’s age before vaccinating the baby, HSA explains to the mother the vaccines that the child will get and identifies by using the route of administration • After administering the oral vaccine, advises the mother to not breastfeed the baby without providing a rationale for that • The mother receives a tetanus vaccine after a nurse checks her health passport book
6 weeks PNC	Facility 3	1	2	• The baby is weighed by HSA and is vaccinated • All mothers are encouraged to keep babies wrapped up until a point of weighing and vaccination to avoid pneumonia • The mother receives her TTV and is advised of the next date of appointment

## Discussion

Immediate postnatal care utilization was high in our study at 91 and 97% among the neonates and women respectively, and this contrasts with findings from the 2019 Malawi Indicators Survey that reported a rate of 88% [5]. Our findings of 6–91% and 14–97% receipt of PNC among women and newborns respectively illustrate an improvement from previous findings in Malawi that showed that overall receipt of PNC services among mothers ranged from 3 to 16% [20]. Our findings further differ from findings that reported low PNC utilization in rural areas and those that reported a higher uptake in urban areas [21, 22], with immediate postnatal care at 88% [5]. The pattern of PNC attendance is striking with more mothers and neonates attending the initial visits, at 91 and 97% respectively, and remains consistent with previous studies where the subsequent postnatal visits had a low rate of attendance [23, 24]. This could be explained by the health system operations where those that received PNC services were likely still admitted to the facility and thus could easily access them compared with the other time points when they must report from home and the attendance at that point could be impeded by some barriers that women face. For Malawi to successfully implement the 4 postnatal visits as advocated by WHO [3], there is a need to revisit the health system operations so that it does not systemically preclude the attendance of the other visits.

While the maternal and neonatal registers review indicates a high receipt of PNC services by 48 h, our observations showed a contrary view whereby we did not observe PNC activities happening during those periods. Our observations revealed a stark reality of the suboptimal care that the participants received encompassing non-receipt of painkillers, minimal levels of counseling on breastfeeding, and scanty neonatal examinations which contradicts the current WHO guidelines and increases the potential of missing critical elements that may indicate complications [3]. This discrepancy could be explained by documentation and reliability challenges with the registers [25, 26]. Our findings from the observations mirror findings from an analysis of Demographic Health Survey (DHS) reports from 33 countries in the Sub-Saharan region that showed that women that attended PNC services in lower-level facilities like the ones we used in this study were rarely assessed postnatally [27]. An analysis of the Malawi 2016 DHS showed that only 48% were assessed by a skilled birth attendant at 42 days which was higher than in our study which was 6% [21]. This could be explained in part by the fragmentation of services at our study sites which are characterized by poor coordination and integration of services that imposes a challenge to attend PNC clinics when women report for immunizations 6 weeks post-delivery [28].

A unique finding in our study was the lack of guidance and proper follow-up and management of orphaned babies and a mother without a living baby; there is a need to provide guidance and training on how to manage these subgroups so that their care is not compromised. There is limited literature on this aspect however a previous study conducted in Malawi on the overall utilization of maternity services showed that women who had a miscarriage were less likely to use health services [29]. This area requires more research so that the experiences of such women and babies are unearthed for optimal and inclusive strategies.

The lack of awareness as reported in our study as a reason for the non-utilization of PNC services was reported in earlier studies in Africa [30–37]. Evidence suggests that most women learn more about PNC during antenatal care services (ANC) thus emphasizing the relevance of education during the ANC period [33]. The lack of awareness expressed in our study could be partially explained by the tendency of women only attend immunization clinics and omit the PNC section [30]. A measure to resolve this problem is an integration of the immunization and PNC services because integration has been shown to improve the accessibility and acceptability of services [38, 39]. Knowledge of PNC can be shared either through health talks within the facility or through mass media [34, 40, 41]. The health promotional messages that are aired in Malawi should extend beyond antenatal care and delivery at a facility and include PNC services as well [40] because women that are knowledgeable of the possible postpartum complications use PNC services [41].

Other barriers to PNC from our study have been previously reported and these include lack of resources [42–44], negative attitude of health care workers [22, 45], cultural values [32, 36] and cultural practices, restrictions, and confinement, and myths during the puerperium [41]. distance [23, 24, 32, 46, 47]. To overcome challenges associated with distance there is a need to incorporate PNC services into the village health clinics and outreach clinics like under-five clinics [42] because this will make them accessible to many within their communities. To realize that goal, health workers that run the community-based clinics will need to be trained for them to provide the required PNC services.

Factors such as education and availability of economic resources as facilitators are consistent with previous findings in Africa that showed that women that were employed and had received a secondary school level education were more likely to attend PNC services [21, 27, 33–36]. Additionally, we argue that women who are knowledgeable and educated would recognize attendance at a PNC as their primary role and responsibility [23, 34–36, 48]. Furthermore, our study highlighted that seeking treatment for an illness facilitates attendance at PNC and it builds upon findings that assert that knowledge of an infant’s danger signals also motivates a woman to attend PNC services with her baby [22, 47]. The realisation that the facility will provide the necessary medical support indirectly motivates the women to attend PNC.

Our findings on the role of male partners in PNC cement previous findings that state that male involvement increased uptake of PNC services [21, 23, 36, 47] possibly because women who are married receive adequate moral and financial support for women to attend PNC services. Previous studies have argued that the absence of spousal support leaves a woman disempowered to decide to use health services for herself and her baby [37, 41]. The gains made in male involvement in PMTCT in Malawi need to extend to PNC so that men are aware that they need to be involved in the entirety of the journey [49].

### Limitations and strengths

Our study had several limitations as follows: Our sample only included those that made use of postnatal care services thereby leaving out insights of those that never accessed the services. Future studies should focus on women and babies that never attend postnatal care so that their perspectives are documented. The use of several approaches for data collection and the triangulation of qualitative and quantitative data are the main strengths of the study because it offers a holistic view for those that ever-used postnatal care.

## Conclusion

Optimization of uptake and utilization of PNC services for mothers and neonates will require the involvement of all stakeholders in the setting. The success of PNC services lies in the communities, health services, and mothers understanding the relevance, timepoint, and services that need to be delivered so that demand for the service is created. Custodians of culture and men need to be involved to mitigate barriers to attendance at PNC. There is a need to assess the contextual factors for a better response in improving the uptake of PNC services and in turn inform the development of strategies for optimizing the uptake of PNC services.

## Supplementary Information

**Additional file 1.** PNC Services Data Extraction Form for the Mother, Version1.0, 01 March 2020.

**Additional file 2.** PNC Services Data Extraction Form for the Neonate, Version1.0, 01 March 2020.

**Additional file 3.** FGD guide for Elderly Women, Postnatal Mothers and Men.

**Additional file 4.** FGD guide for HSAs in English.

**Additional file 5.** English Interview Guide for Health Care Workers.

**Additional file 6.** Observation Checklist- Postnatal Care Services.

**Additional file 7.** Stamped page of consent form.

## Data Availability

The datasets used and/or analysed during the current study are available from the corresponding author on reasonable request.

## Abbreviations

ANC: Antenatal care
FGDs: Focus Group Discussions
PNC: Postnatal Care
