# Supplementary material for: Uptake of, barriers and enablers to the utilization of postnatal care services in Thyolo, Malawi
Source: BMC Pregnancy Childbirth. 2023 Apr 19;23:271. doi: 10.1186/s12884-023-05587-5 (PMC10114368; doi:10.1186/s12884-023-05587-5)
Supplement: Supplementary file 1 — Additional file 1. PNC Services Data Extraction Form for the Mother, Version1.0, 01 March 2020. [file 12884_2023_5587_MOESM1_ESM.docx]

**PNC Services Data Extraction Form for the Mother, Version1.0, 01 March 2020**

| ID Number | Date of Delivery | Place of Delivery | Postnatal Check date | Mother Status | HIV Test | Up to 48hrs | 3-7 days | 8-42 days | HIV Test | Breastfeeding | Episiotomy Tear | Lochia | Uterus | PN Complications | Vit A | FP C | PN FP |
| --- | --- | --- | --- | --- | --- | --- | --- | --- | --- | --- | --- | --- | --- | --- | --- | --- | --- |
|  |  |  |  |  |  |  |  |  |  |  |  |  |  |  |  |  |  |
|  |  |  |  |  |  |  |  |  |  |  |  |  |  |  |  |  |  |
|  |  |  |  |  |  |  |  |  |  |  |  |  |  |  |  |  |  |
|  |  |  |  |  |  |  |  |  |  |  |  |  |  |  |  |  |  |
|  |  |  |  |  |  |  |  |  |  |  |  |  |  |  |  |  |  |
|  |  |  |  |  |  |  |  |  |  |  |  |  |  |  |  |  |  |
|  |  |  |  |  |  |  |  |  |  |  |  |  |  |  |  |  |  |
|  |  |  |  |  |  |  |  |  |  |  |  |  |  |  |  |  |  |
|  |  |  |  |  |  |  |  |  |  |  |  |  |  |  |  |  |  |
|  |  |  |  |  |  |  |  |  |  |  |  |  |  |  |  |  |  |
|  |  |  |  |  |  |  |  |  |  |  |  |  |  |  |  |  |  |
|  |  |  |  |  |  |  |  |  |  |  |  |  |  |  |  |  |  |
|  |  |  |  |  |  |  |  |  |  |  |  |  |  |  |  |  |  |
|  |  |  |  |  |  |  |  |  |  |  |  |  |  |  |  |  |  |
|  |  |  |  |  |  |  |  |  |  |  |  |  |  |  |  |  |  |
|  |  |  |  |  |  |  |  |  |  |  |  |  |  |  |  |  |  |

**Instructions for the PNC Services Data Extraction Form for the Mother, Version1.0, 01 March 2020**

Place of Delivery- Here, Transfer, Home, Other

Mother Status- Alive or Dead

HIV Status- Positive, Negative, Not done

Breastfeeding-Exclusive, Non-exclusive, not-breastfeeding

Episiotomy/Tear- Intact, Infected, Gapped

Lochia- Normal, Heavy, Offensive, Clear

Uterus- Involuted or Sub-Involuted

Postnatal Complications- None, Sepsis, Anaemia, Postpartum Heamorrhage, Severe Preeclampsia/ Eclampsia, Breast Engorgement, Other

Vitamin K- Yes or No

Counselling on Family Planning- Yes or No

Immediate Postpartum Family Planning Method Given- BTL or IUCD
