# Supplementary material for: Uptake of, barriers and enablers to the utilization of postnatal care services in Thyolo, Malawi
Source: BMC Pregnancy Childbirth. 2023 Apr 19;23:271. doi: 10.1186/s12884-023-05587-5 (PMC10114368; doi:10.1186/s12884-023-05587-5)
Supplement: Supplementary file 2 — Additional file 2. PNC Services Data Extraction Form for the Neonate, Version1.0, 01 March 2020. [file 12884_2023_5587_MOESM2_ESM.docx]

**PNC Services Data Extraction Form for the Neonate, Version1.0, 01 March 2020**

| ID Number | Date of Delivery | Postnatal Check date | Complications at Birth | Aspyxiated- Resuscitaed | Baby Status | Up to 48hrs | 3-7 days | 8-42 days | Weight | LBW  KMC | Imunizations | Tetracycline Eye Ointments | Cord Care | Vit K | HEI  NVP | Danger Signs and Symtoms |
| --- | --- | --- | --- | --- | --- | --- | --- | --- | --- | --- | --- | --- | --- | --- | --- | --- |
|  |  |  |  |  |  |  |  |  |  |  |  |  |  |  |  |  |
|  |  |  |  |  |  |  |  |  |  |  |  |  |  |  |  |  |
|  |  |  |  |  |  |  |  |  |  |  |  |  |  |  |  |  |
|  |  |  |  |  |  |  |  |  |  |  |  |  |  |  |  |  |
|  |  |  |  |  |  |  |  |  |  |  |  |  |  |  |  |  |
|  |  |  |  |  |  |  |  |  |  |  |  |  |  |  |  |  |
|  |  |  |  |  |  |  |  |  |  |  |  |  |  |  |  |  |
|  |  |  |  |  |  |  |  |  |  |  |  |  |  |  |  |  |
|  |  |  |  |  |  |  |  |  |  |  |  |  |  |  |  |  |
|  |  |  |  |  |  |  |  |  |  |  |  |  |  |  |  |  |
|  |  |  |  |  |  |  |  |  |  |  |  |  |  |  |  |  |
|  |  |  |  |  |  |  |  |  |  |  |  |  |  |  |  |  |
|  |  |  |  |  |  |  |  |  |  |  |  |  |  |  |  |  |
|  |  |  |  |  |  |  |  |  |  |  |  |  |  |  |  |  |
|  |  |  |  |  |  |  |  |  |  |  |  |  |  |  |  |  |
|  |  |  |  |  |  |  |  |  |  |  |  |  |  |  |  |  |

**Instructions for the PNC Data extraction Form for Neonates Version1.0, 01 March 2020**

Complications at Birth- Well, Asphyxia, Low birthweight/prematurity

Asphyxiated- Yes or no

Baby Status: Alive or Dead

Low birthweight/ Prematurity Kangaroo Mother Care- Yes or No

Immunization- BCG or Polio 0

Tetracycline Eye Ointment- Yes or No

Cord Care (Chlorohexidine used)- Yes or No

Vitamin K- Yes or No

HIV Exposed Infants Nevirapine given- Yes or No

Danger Signs and Complications- none, not able to feed, fever (>37.5^o^ c), Convulsions, Lethargic, Chest In drawing, Fast breathing (>60 breaths per minute) Eye or Cord Discharge, Jaundice
