# Supplementary material for: Uptake of, barriers and enablers to the utilization of postnatal care services in Thyolo, Malawi
Source: BMC Pregnancy Childbirth. 2023 Apr 19;23:271. doi: 10.1186/s12884-023-05587-5 (PMC10114368; doi:10.1186/s12884-023-05587-5)
Supplement: Supplementary file 3 — Additional file 3. FGD guide for Elderly Women, Postnatal Mothers and Men. [file 12884_2023_5587_MOESM3_ESM.docx]

**FGD guide for Elderly Women, Postnatal Mothers and Men**

**Section A -Welcome Remarks**

Agree on group norms such as:

- Respecting each other’s contribution
- Every point is welcome
- Using Pseudo names when talking

Tell me about the common maternal and newborn illnesses in this area.

**Section B - Perceptions on PNC Services:**

1. What is your understanding of postnatal care services?

Probe on:

- What a mother and baby should get and receive as part of PNC
- Beliefs on PNC- individual and communities
- PNC time points for Mother and Newborn

1. What are your roles in PNC services for women and newborns in the community?

**Section C- Barriers to PNC**

1. Explain to me in details the factors that impede PNC services utilization by mothers and newborns in this community?

Probe on:

- Cultural and religious beliefs
- Health System Factors
- Condition of patient
- Community norms
- Orphaned Neonates
- Mothers whose baby died

**Section D- Enablers to PNC Services**

1. Explain to me the factors that enable mothers and newborns to receive PNC services in this community?

Probe on

- Culture
- Health system factors
- Community Factors
- Individual factors
- Occupation – including farming
- Orphaned Neonates
- Mothers whose baby died

**Section E- Strategies for improving provision and Uptake of PNC services**

1. Explain to me the strategies that can be employed to improve provision of PNC services to mothers and newborns in this community?

Probe on:

- Patient Centred Approaches
- Community based approaches
- Facility Based approaches

**NB- For postnatal women FGD- inquire on the PNC services they received, when, where and whom?**

**Section F- Closing Remarks**

1. We are now at the end of the discussion, Is there anything that you would like to add concerning provision and uptake of PNC services among mothers and newborns?

Thank you very much for your time.
