# Supplementary material for: Uptake of, barriers and enablers to the utilization of postnatal care services in Thyolo, Malawi
Source: BMC Pregnancy Childbirth. 2023 Apr 19;23:271. doi: 10.1186/s12884-023-05587-5 (PMC10114368; doi:10.1186/s12884-023-05587-5)
Supplement: Supplementary file 4 — Additional file 4. FGD guide for HSAs in English. [file 12884_2023_5587_MOESM4_ESM.docx]

**FGD guide for HSAs in English**

**Section A -Welcome Remarks**

Agree on group norms such as:

- Respecting each other’s contribution
- Every point is welcome
- Using Pseudo names when talking

Tell me about the common maternal and newborn illnesses in this area.

**Section B - Perceptions on PNC Services:**

What is your understanding of postnatal care services?

Probe on:

- Components of PNC for Mother and Newborn
- PNC time points for Mother and Newborn

**Section C- Barriers to PNC**

Explain to me in details the factors that impede provision of PNC services at this facility and its catchment area?

Probe on:

- Resources
- Volume of Work
- Training
- Capabilities and Knowledge
- Culture
- Operation hours
- Condition of patient
- Operating procedures such as discharge times

Explain to me the patient factors that impede uptake of PNC services at this facility and its catchment area??

Probe on:

- Cultural Beliefs
- Condition of patient
- Community Norms
- Socioeconomic Status
- Education Status
- Occupation – including farming
- Orphaned Neonates
- Mothers whose baby died

**Section D- Enablers to PNC Services**

Explain to me the factors that enable health care workers to provide PNC services at this facility and its catchment area?

Probe on

- Resources
- Volume of Work
- Training
- Capabilities and Knowledge
- Culture
- Operation hours
- Condition of patient
- Operating procedures such as discharge times

Explain to me the factors that enable Postnatal mothers and newborns to take up PNC services at this facility and its catchment area?

Probe on:

- Cultural Beliefs
- Condition of patient
- Community Norms
- Socioeconomic Status
- Education Status
- Occupation – including farming
- Orphaned Neonates
- Mothers whose baby died

**Section E- Strategies for improving provision and Uptake of PNC services**

Explain to me the strategies that can be employed to improve provision of PNC services at this facility and its catchment area?

Probe on:

- Task Shifting Approaches
- Patient Centred Approaches
- Community based approaches
- Facility Based approaches

Explain to me the strategies that can be employed to improve uptake of PNC services by Mothers and Newborns at this facility and its catchment area?

Probe on:

- Task Shifting Approaches
- Patient Centred Approaches
- Community based approaches
- Facility Based approaches

**Section F- Closing Remarks**

We are now at the end of the Interview, Is there anything that you would like to add concerning provision and uptake of PNC services among mothers and newborns?

Thank you very much for your time.
