# Supplementary material for: Uptake of, barriers and enablers to the utilization of postnatal care services in Thyolo, Malawi
Source: BMC Pregnancy Childbirth. 2023 Apr 19;23:271. doi: 10.1186/s12884-023-05587-5 (PMC10114368; doi:10.1186/s12884-023-05587-5)
Supplement: Supplementary file 6 — Additional file 6. Observation Checklist- Postnatal Care Services. [file 12884_2023_5587_MOESM6_ESM.docx]

**Observation Checklist- Postnatal Care Services**

Time points of observations

Follow postnatal clients at 1 hour, 24 hours, 48 hours, one week, and 6 weeks post-delivery.

What to observe

- interactions between postnatal women, and their babies with healthcare workers
- Note the health services they received such as
  - Who provided the service
  - Where service is provided from
  - When a service is provided
  - What is involved in service provision
  - Anything that stood out in the provision of care
- Document existing processes of accessing PNC care
- Identify improvement opportunities
- Outline unnecessary steps
- Highlight waste within the process through duplication of activities
- Identify steps that need re-ordering
- Identify bottlenecks within the process of accessing postnatal care at various points.
