# Supplementary figures and images for: Uptake of, barriers and enablers to the utilization of postnatal care services in Thyolo, Malawi
Source: BMC Pregnancy Childbirth. 2023 Apr 19;23:271. doi: 10.1186/s12884-023-05587-5 (PMC10114368; doi:10.1186/s12884-023-05587-5)

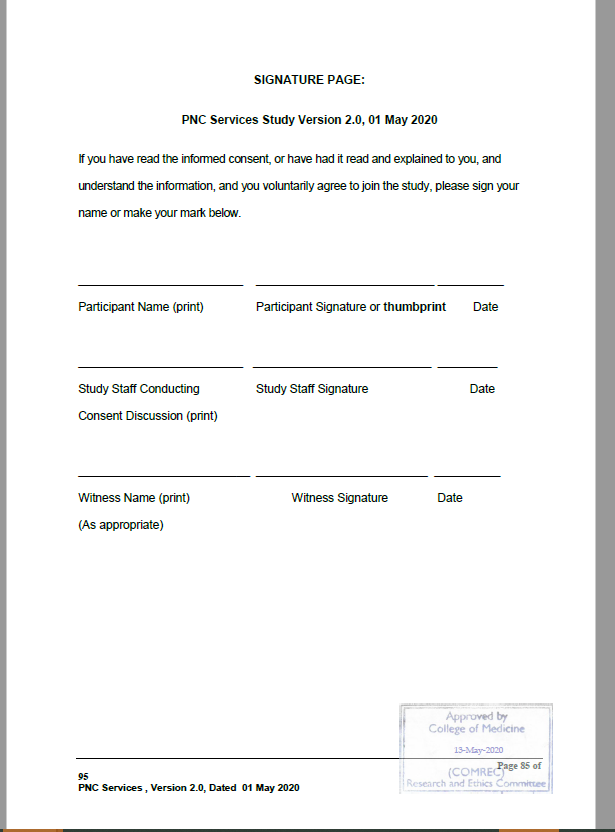

Supplement: Supplementary file 7 — Additional file 7. Stamped page of consent form. [file 12884_2023_5587_MOESM7_ESM.docx]
